# Acceptability and experience of a smartphone symptom monitoring app for people with psychosis in China (YouXin): a qualitative study

**DOI:** 10.1186/s12888-024-05687-2

**Published:** 2024-04-09

**Authors:** Xiaolong Zhang, Shôn Lewis, Xu Chen, Jiaojiao Zhou, Xingyu Wang, Sandra Bucci

**Affiliations:** 1 Division of Psychology and Mental Health, School of Health Sciences, Faculty of Biology, Medicine and Health, Manchester Academic Health Science Centre, The University of Manchester, Manchester, UK; 2 The National Clinical Research Center for Mental Disorders & Beijing Key Laboratory of Mental Disorders, Beijing Anding Hospital, Capital Medical University, Beijing, China; 3 Greater Manchester Mental Health NHS Foundation Trust, Manchester, UK; 4 Advanced Innovation Center for Human Brain Protection, Capital Medical University, Beijing, China

**Keywords:** Psychosis, Remote monitoring, Digital mental health, Acceptability, Feasibility study

## Abstract

**Background:**

Access to high-quality mental healthcare remains challenging for people with psychosis globally, including China. Smartphone-based symptom monitoring has the potential to support scalable mental healthcare. However, no such tool, until now, has been developed and evaluated for people with psychosis in China. This study investigated the acceptability and the experience of using a symptom self-monitoring smartphone app (YouXin) specifically developed for people with psychosis in China.

**Methods:**

Semi-structured interviews were conducted with 10 participants with psychosis to explore the acceptability of YouXin. Participants were recruited from the non-randomised feasibility study that tested the validity, feasibility, acceptability and safety of the YouXin app. Data analysis was guided by the theoretical framework of acceptability.

**Results:**

Most participants felt the app was acceptable and easy to use, and no unbearable burdens or opportunity costs were reported. Participants found completing the self-monitoring app rewarding and experienced a sense of achievement. Privacy and data security were not major concerns for participants, largely due to trust in their treating hospital around data protection. Participants found the app easy to use and attributed this to the training provided at the beginning of the study. A few participants said they had built some form of relationship with the app and would miss the app when the study finished.

**Conclusions:**

The YouXin app is acceptable for symptom self-monitoring in people with experience of psychosis in China. Participants gained greater insights about their symptoms by using the YouXin app. As we only collected retrospective acceptability in this study, future studies are warranted to assess hypothetical acceptability before the commencement of study to provide a more comprehensive understanding of implementation.

**Supplementary Information:**

The online version contains supplementary material available at 10.1186/s12888-024-05687-2.

## Introduction

Access to high-quality mental healthcare remains challenging for people with psychosis globally [1]. Digital mental health, especially smartphone-based symptom monitoring and interventions, have received greater attention in recent years, given their potential to scale up mental health service provision [2, 3]. Conventional symptom monitoring approaches face several practical and methodological challenges, including overly relying on clinic-based face-to-face interviews or comprehensive assessment batteries, which lack ecological validity [4, 5] and the reporting is prone to recall bias [2, 4]. Smartphones can be used to generate a wealth of data through active and passive real-time monitoring methods [2]. Active monitoring is typically defined as a user actively inputting psychological and behavioural data through ecological momentary assessment or other ambulatory assessment methods [5]. Passive monitoring refers to using smartphone or wearable sensors to measure the user’s behaviour or contextual information [6]. Through the integration of both active and passive monitoring, smartphone-based symptom monitoring enables the tracking of a more dynamic, personal, and valid representation of an individual’s emotional and behavioural state [7, 8].

With the unprecedented developments in digital technology in the past decade, smartphones are widely accessible by people with psychosis [9, 10]. In China, around 90% of people with psychosis have access to the internet [11], and people with mental health problems in general are not only willing to use digital health interventions (DHIs) but consider them to be helpful for their recovery [12]. Moreover, evidence shows that smartphone-based monitoring or interventions appear safe to use and are well tolerated by people with psychosis [13–17]. One of the key advantages of digital mental health tools is their ability to increase access to care [18]. The significant shortage of mental health resources affects countries worldwide and has been further exacerbated by the COVID-19 pandemic [19, 20]. China, like many other countries, has been no exception to this challenge. The estimated shortage of mental health professionals in China relative to the population need was 40,000 [21]. Despite the efforts China has put into improving the accessibility and quality of mental health services in the past decades, the outcome has only been partially successful and the gap remains significant [22, 23]. In China, mental health services, including the treatment and management of psychosis, are predominantly provided by public psychiatric hospitals and supported by psychiatric units in general hospitals, community-based health facilities and rehabilitation centres [24, 25]. Outpatient clinical visits at psychiatric hospitals tend to be scheduled mostly monthly, and unlike most mental health systems in Western countries, a care co-ordinator is not available in China [26]. Moreover, most well-trained mental health professionals are concentrated in psychiatric hospitals located in urban areas [27], as mental health services are primarily delivered through psychiatric hospitals in China [24, 25, 28], which makes it difficult for people in rural or remote communities to access care [29, 30].

Digital mental health holds enormous promise to address the challenges mentioned above, including overcoming some of the barriers faced by conventional symptom monitoring approaches [2] and the shortfall of mental health resources in China [31, 32]. Several studies exploring digital mental health have been conducted in China; for example, smartphone apps have been developed to prevent postpartum depression [33], facilitate training for children with autism spectrum disorder [34], support drug addiction [35], promote well-being in the general population [36], and establish ecological momentary assessment for depression [37]. Despite its potential, the generally low real-world usage rates remain a key obstacle to realising the full potential of DHIs in psychosis [38]. Users attitudes and beliefs about interventions and the complexity of interventions are key factors affecting implementation of DHIs [39]. Therefore, an in-depth understanding of users expectations, views and experiences of using DHIs in both the development and the evaluation phases of DHIs is needed to maximise user engagement [40, 41].

Although smartphone-based symptom monitoring in people with psychosis has proved to be feasible in the Western context [42], to date, no such tool has been developed and evaluated for people with psychosis in China [31]. To address this gap, we co-produced with service users and staff the YouXin app, a symptom self-monitoring smartphone app for people with psychosis. This study aimed to explore the acceptability of YouXin and the experience of using the app through interviews with people who participated in the larger feasibility study evaluating the validity, feasibility, acceptability and safety of the YouXin app [43].

## Methods

### Study setting and participants

This qualitative study was nested in the YouXin non-randomised feasibility study of a smartphone symptom self-monitoring app for people with psychosis in China [43, 44]. As the data generated from the qualitative interview was rich, we were not able to give it full justice in the main outcome feasibility study paper. Therefore, we reported the findings in their own right in order to give a full account of the data. Participants were recruited from the outpatient department of Beijing Anding Hospital of Capital Medical University, a major tertiary psychiatric hospital in Beijing, China. Participants were asked to use YouXin to self-monitor their psychotic and mood symptoms over four weeks. At the end, the research team invited participants to take part in a qualitative exit interview. Of the 40 participants who participated in the non-randomised feasibility study, seven were lost to follow-up, 23 were not interested in taking part in the interview, and 10 took part in the post-intervention interviews. Eligibility criteria of the YouXin study were deliberately inclusive to assess feasibility and acceptability of the app. Inclusion criteria were: (1) diagnosed with a DSM-5 (F20-F29) schizophrenia spectrum disorder or met criteria for being at clinical high risk for psychosis according to the Structured Interview for Prodromal Syndromes (SIPS); (2) aged 16 to 65; (3) receiving care from Beijing Anding Hospital, China and continued to be actively supported by the hospital for the trial duration (5 weeks); (4) owned and able to use a smartphone; and (5) clinically stable as judged by the responsible clinician. Exclusion criteria were: (1) diagnosed with organic or substance induced psychosis; (2) lack of capacity to provide informed consent as judged by the responsible clinician; and (3) judged to be at risk for self-harm or harm others.

### Intervention

YouXin is a smartphone app-based symptom self-monitoring tool designed for people with psychosis. The app is adapted from the ClinTouch app [45]. To develop the YouXin app, we selected the validated ClinTouch psychotic and mood symptom items and translated the items into Chinese. We added a passive monitoring function to the app to extract objective mobility and activity indicators to primarily assess negative symptoms. The YouXin app was developed by a multidisciplinary team, including academics, clinicians, software engineers and experts by experience. We adopted a systematic co-production approach to development, aiming to optimise usability and acceptability from both service user and clinician perspectives. The core functions of YouXin consist of active monitoring of current symptoms (termed ASM; including psychotic and mood symptoms and contextual information) in near real-time and passive monitoring of behavioural activity (i.e. Global Positioning System, GPS; step count). Examples of ASM items are shown in Table 1. The ASM prompts were set to alert at two pseudo-random timepoints per day in a 12-hour interval from 10:00 am to 10:00 pm seven days a week for four weeks. This prompt frequency was set to minimise participant assessment burden. Participants could snooze the prompt for 5 or 30 min or decline to answer. GPS and step count data were passively collected from smartphone sensors to measure users’ mobility and activity levels. A line chart will visualise the results of monitoring based on the data entered into the app. The screenshots of the YouXin app are shown in Supplementary Fig. 1.

**Table 1:** Examples of the ASM items

Domain	Item	Response items	Format
Psychotic symptoms	‘I have heard voices’	1–7	Likert scale
Mood symptoms	‘I have felt sad’	1–7	Likert scale
Contextual questions	‘Where were you?’	At home; at work; at school or other educational setting; in hospital; in vehicle; in convenient store, supermarket or shopping mall; outside walking; inside other; outside other	Check box

Participants were given a training session to help them navigate the app. A member of the research team supported the download and installation of the app onto a participant’s smartphone and demonstrated the app. Participants could practice using the app and ask questions during the process. We encouraged participants to use the app from the training session until the 4-week assessment timepoint. We implemented an opt-out approach, allowing participants to turn the passive sensing on or off at their discretion throughout the study. Participants were informed at the training sessions that the app was not suitable for seeking urgent medical care. Further details of the study procedure can be found elsewhere [43].

### Procedure

Participants were informed about the exit interview when the research team contacted them to participate in the YouXin study. Consent was obtained together with the larger feasibility study. All participants who consented to the feasibility study were invited to the semi-structured qualitative interviews at the 4-week follow-up meeting. Participants were interviewed in person or remotely per their preference. Participants received a 100 RMB (approximately £10) gift voucher at the end as a thank you for their time and to cover any costs incurred due to their participation. An interview topic guide was designed based on our own research group’s previous work. It aimed to explore the feasibility and acceptability of using the app, experience of participating in the study, and potential barriers and facilitators to the app’s implementation. More specifically, topics covered were: (a) overall impressions of YouXin; (b) positive and negative aspects of the app in terms of content and usability; (c) how it helped and/or did not help; (d) what changes they would make; (e) barriers and facilitators to engagement; (f) views on the training session. The topic guide is shown in Supplementary Table 2.

### Analysis

The interviews were conducted in Mandarin Chinese. To enable the larger research team to analyse and interpret the data, audio-recordings were transcribed verbatim in Chinese and translated into English by XZ. Back translations were performed by XW independently, and then XZ and XW met to discuss the accuracy of the translations until an agreement was reached. The finalised English transcripts were then imported into Nvivo 12 software [46]. Data were analysed following Braun and Clarke’s [47] thematic analysis approach, which consists of six phases: familiarisation, transcription, generating initial codes, searching for themes, reviewing themes, and then defining and deciding on meaningful themes. The analysis was also guided by the theoretical framework of acceptability (TFA), an established multi-construct theoretical framework of acceptability of healthcare interventions [41]. This framework comprises seven constructs: affective attitude, burden, ethicality, intervention coherence, opportunity costs, perceived effectiveness, and self-efficacy. After open-coding the transcripts, the theme ethicality was deemed irrelevant to this data; therefore we coded the data into the remaining six TFA constructs (Table 2). One additional theme, privacy, was added. Data regarding how YouXin can be implemented and improved was coded and reported separately.

**Table 2:** Thematic framework adapted from the theoretical framework of acceptability

Construct	Description
Affective attitude	How the participant feels about YouXin.
Burden	The perceived amount of effort that is required to participate in YouXin.
Intervention coherence	The extent to which the participant understands YouXin and how it works.
Opportunity costs	The extent to which benefits, profits or values must be given up to engage in YouXin.
Perceived effectiveness	The extent to which YouXin is perceived as likely to achieve its purpose.
Self-efficacy	Participant’s confidence that they can perform the behaviours required to participate in YouXin.
Privacy	The extent to which the participant feels comfortable sharing personal information and data with YouXin.

## Results

Ten participants took part in the interview. Participant demographic and clinical characteristics are shown in Table 3. The median age was 29 (IQR = 21–41). Most participants were male, Han Chinese, and received a diagnosis of schizophrenia. All the participants received antipsychotic medication. Interviews lasted from 16 to 45 min. The demographic and clinical information for each participant are summarised in Supplementary Table 1.

**Table 3:** Demographic and Clinical Characteristics: Numbers (%) of Participants

Characteristics	Values
Age, years– median (IQR)	29 (21–41)
Gender	
Male	7 (70)
Female	3 (30)
Ethnicity	
Han	9 (90)
Mongolian	1 (10)
Education level	
High school	3 (30)
College	4 (40)
University	3 (30)
Employment status	
Full time	4 (40)
unemployed	4 (40)
student	2 (20)
Diagnosis	
Brief psychotic disorder	2 (20)
Clinical high risk	1 (10)
Schizophrenia	7 (70)
Medication(s) used	
Antipsychotics	10 (100)
Antidepressants	2 (20)
Anti-anxiety medications	3 (30)

### Theme 1: affective attitude

Most participants felt positive about participating in the study and using the app. Moreover, completing the monitoring gave them a sense of achievement:

“There is a sense of joy in answering the questions, a sense of joy and then you feel quite a sense of achievement… then you feel motivated to do things” (Participant 1).

“The overall feeling is, ah, every day I answer the questions as soon as the alarm clock goes off, and I feel quite fulfilled, I feel like I’m helping people do research or something, and I’m quite fulfilled to finish answering the questions” (Participant 21).

More specifically, some participants described feeling good about being more aware of their symptoms, a sense of achievement when completing the item set, and a sense of fulfilment that when they could see that their symptoms appeared stable:

“Especially after I finish the second set of questions at night, I feel that my state is very stable and good today, and I feel a sense of achievement and joy, I feel good, that’s the main thing” (Participant 1).

Many participants reported they liked the design features of the app and found the content of the app relevant to their mental health condition:

“My feeling is that the interface is quite new, gives people the feeling that the software is quite newly developed, quite similar to the app I usually use… what I like is that I can change the avatar and the background of the home page, I like that” (Participant 21).

“At first glance, I felt that it was quite right for my situation, ah, for my condition, and then, it was quite relevant” (Participant 9).

In contrast, some participants felt the app was less meaningful for them. In particular, the repetitiveness of the ASM items and the simplicity of the app functionality decreased some participants’ motivation to engage in or use the app in a sustained way:

“It’s like this is what I do every day anyway, my work and life is like this, just record it, it also does not matter if you record it or not” (Participant 3).

“The same few questions every day, no change… it doesn’t really fit in, it can’t socialise with other users, the app is a single-player game” (Participant 14).

“I feel that everything is like this, just when I first started to do it, I was quite punctual, but later… because it is always repeated, every day repeat the same questions, so I sometimes may not be particularly serious, at the end may be a little bit like this” (Participant 18).

When asked if they would miss the app at the end of the study, some participants indicated that they got used to having the app on their phones, that they felt a sense of attachment and connection with the app, and that they would miss it when they were no longer able to access it:

“It’s true that I miss it a little bit… I didn’t want to uninstall it, I still want to continue to use it, I think it’s quite good, yes, it’s similar to a kind of life punch card” (Participant 1).

“It gives me the same feeling as a doctor… it is a sense of attachment [laugh]” (Participant 5).

However, some participants did not have any strong feelings about deleting the app and losing access at the end of the study. Instead, some participants described feeling relieved since they did not have to think about answering the question set in the app, and they couldn’t necessarily see a direct impact on how completing the items might support their care:

“There is no impact, answer it or do not answer it, it does not matter, anyway it is just a kind of game, if I don’t need to answer it again, it seems to be a little easier [laugh], I do not have to do a task” (Participant 3).

“I probably don’t miss it particularly… I probably feel temporarily like there’s one less thing to do, like it’s a bit more relaxed” (Participant 21).

Of note, one participant reported that it felt uncomfortable to give “truthful” answers in response to ASM questions in the event their treating clinician will view their responses or they themselves will have evidence of possible deterioration in their mental health:

“There are some questions that are, I’m afraid to, umm, dare not express some of the real thoughts, for example, whether paranoid, then I will think am I paranoid today, just kind of let myself think back to these things, but I do not dare to fill in the accurate answer… for example, it has reached 7 but my response is 3, just like that… it’s not exactly hiding, but I’m just afraid to answer the most accurate one anyway… maybe because I will think of the doctor will look at my answers, or if I give a real answer, I can see the parameters of the data, ah, every time it reaches three I will feel pressure, yes, maybe it is about this aspect of it… it’s too stressful, that’s what it feels like” (Participant 1).

### Theme 2: Burden

Participants overall found the effort they made to engage in the study and the app acceptable and that involvement was not too intrusive or effortful:

“Well, it was okay, not too intrusive, the processes were actually quite fast” (Participant 4).

“Well, no, I just finished it easily, it took one or two minutes” (Participant 5).

Some burden was reported by participants; however, participants did not report this sense of burden to be distressing or intolerable. A few participants felt that the two randomised prompts a day were unnecessary and the randomness with which these were sent was problematic as they preferred to know when the prompts would be sent so they could plan their day better. Participants also said that the repetitiveness of items was problematic:

“Well, that time is always random… it’s twice a day, it’s not the same time, sometimes you can’t catch up and it’s over, why can’t it be fixed time, why it has to be random” (Participant 14).

“I actually don’t think that twice a day is necessary, I think once a day is about right, and then the questions, well, it’s sometimes repetitive… I don’t know how to improve it, but, just, it’s true that in the process of answering the questions, well, some of them are a bit problematic… it’s the same question it always repeats” (Participant 18).

Burden related to technology issues were reported, including setting one’s own alarm clock to complete the ASM questions set due to the alarm volume of the app being too soft, and the need to charge the phone more often than usual:

“It’s just that sometimes I can’t remember, so I end up setting an alarm clock, and answering the questions as soon as the alarm goes off 5 minutes earlier… its [own] alert volume is too small, sometimes you can’t hear it, it is too small” (Participant 19).

“So the power consumption is a bit more, the phone loses power… before I use this app, I charge the phone once a day and with no problem, now it will run out of power at 6 or 7 pm, 7 or 8 pm… [but] it doesn’t matter too much, because you can recharge at lunchtime too” (Participant 21).

Regarding burden related to study procedures, all participants felt the study procedures were acceptable, except one participant who reported that participating in the study was time-consuming:

“I spent much time on it [laugh], so I can’t do other things… It does take some time, some time costs” (Participant 3).

### Theme 3: intervention coherence

All participants reported the YouXin app was easy to use, understood how it works, and its content was relevant to their mental health condition. However, a small number of participants found some of the terms in the ASM items and the results graph difficult to understand:

“It has the word ‘grandiosity’, and I’m not quite sure what this ‘grandiosity’ means, that’s what I don’t quite understand” (Participant 18).

“I don’t know how to read the results, because that line [graph], that curve, I don’t know how to read, it overlaps in many places, just doesn’t look good at a glance, it would be better if it can be presented more straightforward” (Participant 19).

The training provided at the beginning of the study was deemed useful by all participants. More specifically, participants felt the training gave them detailed information on how to operate the app:

“It gave me some ideas to better complete this thing, if you do not tell me this, it will cost me a long time to figure it out, after the introduction, I can use the app easily, or I have to figure it out myself” (Participant 3).

A small number of participants reported, however, errors in the app when they recorded their response to ASM items or when passive data was recorded:

“I finished this question, and then I tried to review the previous question, but the answer it showed me was not the answer I gave” (Participant 18).

“It’s the step counting, it is not very clear… sometimes it doesn’t record when I walk, but sometimes it does” (Participant 5).

### Theme 4: opportunity costs

In general, participants indicated that the YouXin app fit well in their daily lives, and participants did not need to significantly sacrifice aspects of their time or day to engage in the app and the study procedures. On occasion, some participants said that they missed responding to alert notifications that clashed with other aspects of their routine or when they forgot to take their phone with them:

“Actually, I don’t think it affects me very much, but I think for some work or some family gatherings or something, for example, if I miss it, or if I can’t answer the question at that time, it will have a reminder for 20 minutes later, but if you don’t click on the reminder for 20 minutes later, it will miss the question, I feel like I haven’t finished the question again today” (Participant 1).

“Just don’t put it too late to answer the questions, I was sleeping at that time so I missed a lot of prompts… after 9 o’clock, I think, I go to bed quite early” (Participant 5).

“I remember just a few times I forgot to take the phone and didn’t do it… I didn’t take the phone for a while, so I missed one or two, I can’t remember, maybe because I was doing something else” (Participant 2).

Some participants said that they prioritised responding to the app over other activities, but this did not have any negative consequences on their day-to-day life and did not cause distress:

“Basically, if I was busy at the moment, I just put things aside for the time being, the app doesn’t take long, just one or two minutes, two or three minutes to answer, after answering [it] I’ll continue to do my things… it just a couple of minutes, it doesn’t matter” (Participant 3).

“Sometimes, for example, when I want to take a nap in the noon or afternoon, I have to think of that there is an alarm clock that will go off later, so I may not be able to sleep for too long” (Participant 21).

### Theme 5: perceived effectiveness

All participants agreed the app achieved the goal of symptom self-monitoring. Specifically, participants found that answering the questions facilitated self-management of their mental health:

“What is helpful is that it will detect whether you have that strange thought, for example, if I have a relapse, and then when I answer the questions, the delusions score may be quite high, it may be able to remind me that I may have a relapse soon, and I may need to go to the doctor as soon as possible… I feel like I have some sense of control over my mental health, that I feel like I’m in control, I know if I’m going to have a relapse, I have that sense of control” (Participant 21).

Responding to ASM items supported participants to better understand and communicate their mental health with others, including their doctor and family members:

“The questions, I think the questions are quite comprehensive, and it’s also quite straightforward, well, some of the questions it reflects directly, like, it might be like after you answering this question you will know what aspect it reflects” (Participant 18).

“It helps the doctor to treat me [laugh]… just show her the results straight away… it’s convenient, because there are things I can’t describe clearly, but the assessment is straightforward… it just doesn’t help me with my specific condition, but it’s nice to be able to communicate with my doctor” (Participant 5).

Passive monitoring provided feedback on mobility and activity level and served as a reminder to participants to exercise, which resulted in positive effects on both mental health and physical health:

“I check the step count every day, so I know whether I’ve exercised today, and then if I am on rest and not exercising, I’ll tell myself get up and walk, don’t stay still” (Participant 1).

“This passive monitoring… it is good for the patient to be more active, strengthen the physical well-being, strengthen the immune system, and then enhance confidence… I think this physical exercise is particularly good for the treatment of this condition [psychosis], and it has a positive treatment effect” (Participant 19).

However, a few participants felt the app was not helpful because they were not particularly symptomatic and therefore the questions did not seem relevant at the time. Also, the repetitiveness of the ASM items meant that participants felt they were responding to questions not relevant to their situation repeatedly:

“The symptoms that I have had before, some of the symptoms are in the app, but I experience none of them at the moment, so the app is not too useful, my symptom was quite severe before, back then, it would be more useful, now it seems I am normal as I take medicine and other treatments, so it is not particularly helpful to me anymore… it doesn’t mean much to me, it is useful for people who have some symptoms, so that it can record fluctuations in a certain way, for example, today the score is 1, and tomorrow maybe 2 or 3, it is more useful for people experiencing symptoms” (Participant 3).

“Because the questions generally, um, it seems that there are not many changes in each day’s questions, all my answers were absence of symptoms, so I do not feel the app has any effect on me” (Participant 21).

Additionally, participants indicated that the app was useful when in-person mental health services were unavailable, such as during the COVID-19 pandemic, but felt that digital tools cannot replace face-to-face mental healthcare:

“If it is under a situation like the pandemic, it is not convenient to see a doctor, then this is helpful, but it is still not as good as seeing a doctor face-to-face, face-to-face is more effective and better” (Participant 19).

### Theme 6: self-efficacy

Participants were confident that they could operate the YouXin app and found the user interface and contents of both the ASM and passive monitoring components straightforward. Some participants reported they were more proficient using the app as they became more familiar with the content:

“The change is probably that the speed of answering the questions is a little bit faster, well, there are questions that I know are three or four questions in a row, and then I know that the first choice, for example, if I chose no delusion, and the next three should all be score 1, and then I answer it very quickly” (Participant 21).

Though participants themselves were competent in using the app, concerns were expressed that it might be difficult for some populations to use the app effectively, such as people who are not so familiar with digital technology, and children:

“There is something I don’t like is that it may need to set some parameters on the Android phone, for example, you need to turn on some permissions or something like this when you are recording steps, I think I am able to set this thing in the background, but there are some people, for example, they may not know much about electronic products, they may feel difficult to set it up” (Participant 1).

“But some patients are not easy to manage themselves very obediently [laugh]… for example, children… a child self-manage mental health condition through an app is quite difficult to achieve” (Participant 5).

### Theme 7: privacy

Participants were not concerned about privacy and data security issues, mainly as they had been informed what data would be collected in the consent and training session and because they reported an inherent trust in the hospital in relation to data protection. Participants also reported that conducting this type of research was consistent with the hospital’s role and remit:

“I have been clearly told that it will monitor some of my data in the training, so I already knew that before, so I think it is not a big concern” (Participant 1).

“Because I know that my steps and records and stuff will not be leaked to any strange people, I know you will add privacy protections, so it is fine, so I didn’t feel that way” (Participant 21).

“Security, privacy, no, no worries, because the hospital is meant to study the disease with patients, and then it can benefit you, so I cooperate with the hospital to do research” (Participant 9).

Despite the sensitive nature of GPS data, participants felt that it was acceptable and appropriate to record their location data in the app:

“No, no, I think the privacy is similar to you answering questions” (Participant 18).

“I didn’t care too much of it, just always on, and I didn’t feel being monitored all the time, it’s just very natural” (Participant 21).

Although no concerns were raised regarding privacy, participants mentioned the importance of informing users about privacy issues:

“Well, it’s still the privacy issue, maybe, yeah, if you do not tell me exactly why I’m answering this question, I would be concerned about the privacy issue, so I won’t tell the most accurate and direct answer, I will provide an ambiguous [answer]” (Participant 1).

Some participants were comfortable using the app when other people were around as they perceived it to be similar to other apps they usually use. Additionally, some participants said the question set could be completed quickly, so they felt people around would not notice what app or content they were interacting with. However, some participants expressed their discomfort at others knowing what content they might be interacting with in the app, mainly due to the stigma associated with mental health problems:

“I won’t tell them, and if I have to tell them when answering this question, I will just say it is a learning app so I need to answer some questions, and they will not pay special attention to what you are doing, anyway it is very simple to complete, but I will not open the conversation and tell them that I am using a software [for mental health purpose]” (Participant 1).

“[If] they see me answer [the app] and ask me what I am answering, how can I explain this to them, [if] I say I am doing research and they will look at me differently… it’s better to answer by yourself, so no one can read it” (Participant 14).

“I just don’t want other people to know that I have this disease, nowadays society is still discriminating against this kind of disease, but it’s getting better and better, and sometimes you can’t hide it even if you want to… basically I just let it be” (Participant 19).

### Suggestions for implementation and improvement

Participants’ willingness to use the app in the future and their perceived barriers and facilitators of implementation were explored (see Table 4). Overall, participants were willing to use the app in the future, anywhere from two weeks until recovery. Regarding barriers to implementation, the popularity of digital mental health apps, access to smartphones, family member/carer opinion, symptom severity, and burden to engage in the app were identified. Participants considered doctor recommendations and advertising in hospitals (e.g. posters) could facilitate the use of the YouXin app in the hospital.

**Table 4:** Perceived barriers and facilitators of implementation

Themes	Representative quote
**Implementation: Barriers**	
Access to smartphone	*“Some patients can’t carry mobile phones… mobile phone is not allowed to be used when hospitalised, you can only use the public telephone, so that is more difficult, people can only use the computer in the computer room to answer questions, so this may have some influence, because no mobile phone, not allowed to bring mobile phone, may have some influence” (Participant 2).*
Burden of engaging in the app	*“I think one of the obstacles is that the patient may be too bothered to fill in the form every day, and another is that the patient may be too bothered by the power consumption of the phone and the app” (Participant 21).*
Family member/carer opinion	*“Parents may not like their children or do not like patients to use it, they may feel that it may trigger patients to think about their illness or something… And the parents themselves don’t accept it very well” (Participant 5).*
The popularity of digital mental health apps	*“One [barrier] is that people nowadays are used to playing games and watching TikTok, but it is difficult for them to accept a software that is not popular and to adapt to this software and use this software, I think this is a factor, but when everyone is using TikTok, for example, in a circle when everyone is using the app then I think people will follow, because it’s also a benefit” (Participant 1).*
Symptom severity	*“Unless the person is with serious illness, he can’t take care of his own life, he can’t answer the questions, well, people with mild or moderate conditions can complete this” (Participant 19).*
**Implementation: Facilitators**	
Advertising	*“Like posters, posters that ask people to join the project, I will occasionally look at such posters, so I may choose to participate” (Participant 21).*
Mental health professionals’ recommendation	*“The doctor’s direct recommendation to the patient is the best and most acceptable, and the patient listens to the doctor the most [laugh]” (Participant 19).*

Suggestions for improvement to the app, the training session, and the study procedures were collected. Many participants suggested adding more ASM items to cover more symptom domains, including sleep problems, cognitive function, and mood symptoms in a specific environment (e.g. feeling anxious when walking in a crowded environment). Contradictory suggestions on the number of questions were identified; some participants wanted fewer items so that it took less time to complete the ASM question set, while others suggested adding more items so that more symptom domains could be explored.

“Just make the questions a little less, a little more precise, add sleep to it… other than that it’s fine, just fewer questions, so it doesn’t have any impact on life or anything” (Participant 3).

“You can add some more, I think the number of questions is still a little bit small, for example, say anxiety, you can have 5 to 7 questions like this kind… and then, for example, sleep disorders or something, emotional instability, also add 5 to 7 questions, now the number of questions is a little bit low” (Participant 19).

A few participants suggested fixing the alert notification schedule to a particular time (rather than notifications being sent pseudo-randomly) or increasing the snooze period so that they could respond at a time that suited them, which would in turn reduce the chance of prompts being missed. Some participants also said that they would have liked the app to provide personalised advice for their mental health following responses they made to the ASM item set and their daily entries. Participants suggested modifications to line graph are needed to so that it is easier to read; the overlapping lines representing different symptom domains was too complex to meaningfully interpret for some. Participants proposed adding some new functionalities to the app, including gamification to make it more rewarding to engage with, fitness tracking, relapse prevention strategies, and a photo diary.

“I’d like to have some pictures, like, I’ve seen something good recently, I’d take a picture of it and add a picture of my mood, pictures and text are important nowadays, just plain text is a bit too boring… for example, if you ride a bus today, take a picture of what you’ve seen, or where you’ve been, take a picture” (Participant 14).

Regarding the training session, a few participants expected the researcher to provide more detailed information about the rationale and purpose behind the ASM items and the app so they could have a deeper understanding of the items and their own symptoms. Finally, participants indicated that the study procedure was well-designed and no improvements to this was needed.

## Discussion

The aim of this study was to explore the acceptability of the YouXin app and how participants experienced it. Most participants felt the app was acceptable and easy to use. Participants found completing the self-monitoring rewarding and experienced a sense of achievement. Most participants tolerated receiving two prompts per day, and no unbearable burden or opportunity costs were reported. Privacy and data security were not expressed as a concern by participants largely due trusting the hospital’s data protection procedures. Nevertheless, some participants preferred to answer the app alone without other people around for reasons related to stigma associated with mental health problems. The app was considered helpful in symptom self-monitoring, making participants more aware of their symptoms, and better communicating their mental health with mental healthcare providers and family members. All participants found no difficulties using the app; some participants attributed this to the training provided at the beginning of the study. Several participants said they had built some form of relationship with the app and would miss the app after completion of the study. Nonetheless, some improvements were suggested by participants, including optimising the alert nonfiction schedule for better time planning and fewer missed prompts, adding more symptom domains in ASM items so the app can be more meaningful for people wanting to monitor mental health conditions other than psychotic and mood symptoms, refining result graph layout with textual advice to enhance comprehension, and expanding the functions of the app to make it more engaging.

Effective symptom monitoring is arguably a critical element for high-quality mental health services. However, due to the limited mental health resources in China, timely symptom assessment is not widely accessible [23, 27]. Most participants emphasised the app increased their awareness and deepened their understanding of their symptoms, subsequently aiding them in making adjustments to their daily life and putting strategies in place to help manage their mental health. The app was described as a platform to help participants communicate their mental health condition with both mental health professionals and family members. Many participants reported that, before using the app, they did not have the means to measure their symptoms and sometimes found it difficult to communicate their exact feelings to their healthcare professional as they felt they did not have the language (terminology) or ability to express how they were thinking/feeling. These findings support the idea that using smartphone apps to self-monitor symptoms is acceptable and beneficial for people with psychosis and serves a purposeful function [14, 45, 48]. However, as participants mentioned the line graph displaying their symptom reports was difficult to understand, refinement is needed support users to interpret graphical read out of symptom monitoring and ensure meaningful comprehension of their mental health condition.

Perceived level of usefulness of YouXin seemed to be a factor that influenced the extent to which participants engaged with the app. For example, a few participants with milder symptoms expressed that the monitoring results did not change or fluctuate over the app exposure window and so they felt it had little utility, resulting in the feeling less motivated to use the app over a longer duration. Additionally, the repetitiveness of the ASM items and the simplicity of functionality for some led to disinterest. Boredom and fatigue effects caused by the repetitiveness of items has also been reported in previous digital remote monitoring studies [13, 48]. Some participants suggested adding gamification elements to make self-monitoring health apps more engaging and rewarding. Moreover, some participants proposed adding more functions to the app to further enhance the user experience and promote meaningful engagement with the content, including fitness tracking, a photo diary, and relapse prevention strategies. As complex smartphone-based self-management tools have been shown to be feasible and beneficial [13, 15, 16], updates for the YouXin app are needed to adapt more elements to expand its functions to meet the broader needs of users and maximise longer term engagement.

Most participants felt passive monitoring was acceptable and non-intrusive, with many participants describing that they were comfortable to leave passive data collection running in the background without actively attending to it. This finding is in contrast to some studies that have found that people with psychosis might feel paranoid about routine monitoring in the context of their healthcare [17, 49–52]. While non-intrusiveness is viewed as positive from an acceptability perspective, a downside of passive monitoring is that the smartphone’s operating system may halt background data collection for performance or battery reasons when users do not interact with the app after an extended period, without sending any notification [53]. This fact may explain the high rates of missing data for passive monitoring found in the YouXin study [54], and in other passive monitoring studies [53, 55, 56]. In addition, participants mentioned power consumption was higher than before using the app, which is likely caused by passive monitoring [57]. Therefore, optimising passive data collection strategies to reduce missing data and power consumption is needed.

Unlike previous qualitative studies [58–60], we found participants were not overly concerned about privacy and data security despite the intense collection of data by the app. Most participants said it was because they consented to the study and trusted the hospital around data protection. This finding suggests that, within the context of Chinese tertiary psychiatric hospitals, if service users put trust in the hospital, it alleviates concerns around privacy and data security issues, thereby not hindering people from using the technology. Trust and transparency are core elements for successful implementation of digital mental health [18, 61], especially for symptom monitoring tools which often requires sharing a considerable amount of data [62]. Building trust requires adequate governance and regulation [63]. Despite lagging behind in the regulation of digital health compared to most Western countries, China has recently launched a policy on internet healthcare aiming to regulate and strengthen the development of the online healthcare system [64]. Nevertheless, further efforts are needed from regulators in China to develop policies specific to digital mental healthcare to foster a healthy, transparent, and trustworthy development environment.

Most participants found the app seamlessly integrated into their daily lives, and some participants described a sense of connection with the app and would miss it when the study stopped. This reflects the concept of Digital Therapeutic Alliance (DTA), which describes the therapeutic alliance built between the users and a digital mental health app [65]. Even though the YouXin app was a stand-along automated app with no active human support, we found participants reported establishing a connection or bond with the app, in line with Tong et al’s [66] finding that DTA could be built between users and a fully automated mental health app. Although DTA is a relatively new concept [67], our finding underscores the importance of considering DTA in the development of mental health apps. As therapeutic alliance has shown to be important in predicting outcomes in traditional psychological therapy, we expect DTA might have similar effects on DHIs.

### Strengths and limitations

To our knowledge, this is the first study to use qualitative interviews to explore the acceptability of symptom self-monitoring using digital tools in people with psychosis in China. We systematically examined the acceptability of the YouXin app, the training session, and the study procedures, gaining insights into participants experiences on the app and the study. We also identified barriers and facilitators that may influence the implementation of digital mental health apps in people with psychosis in China.

There are some limitations of this study. Participants were recruited from the YouXin feasibility study and owned a smartphone. Therefore, our sample may have had higher digital literacy and interest in digital health compared with the broader psychosis community in China. Moreover, we did not explore acceptability before and during the study and only collected retrospective acceptability at the end of the study. This limited our understanding of the hypothetical acceptability of people with psychosis on the app, which is increasingly considered an important factor in fully understanding the implementation of digital mental health [40, 68]. Future studies should explore participants’ views prior to the commencement of the study to be able to compare and contrast acceptability before, during and after the intervention, which has the potential to provide a more comprehensive understanding of implementation factors to be considered when integrating digital health interventions into routine clinical practice.

## Conclusions

This qualitative study indicates the YouXin app is acceptable for symptom self-monitoring to people with psychosis in China. Participants gained greater insights about their symptoms by using the YouXin app, while suggestions for improving user experiences, functionality, and perceived effectiveness were reported. We found some form of DTA was developed between participants and the YouXin app, suggesting more comprehensive investigations on DTA are needed, especially for long-term symptom monitoring. We only collected participants views at the end of the study; future studies are warranted to assess acceptability before and during the commencement of study to provide a more comprehensive understanding of implementation factors. This study not only informs the refinement of YouXin, but also provides valuable information for the development and implementation of other DHIs for psychosis in China more broadly.

### Electronic supplementary material

Below is the link to the electronic supplementary material.

**Supplementary Material 1:** Supplementary Figure 1. Screenshots of the app prototype, Supplementary Table 2. Interview Topic guide

## Data Availability

The data sets generated during or analysed during this study are available from the corresponding author on reasonable request.

## Abbreviations

DSM-5: Diagnostic and Statistical Manual of Mental Disorders, Fifth Edition
SIPS: Structured Interview for Prodromal Syndromes
ASM: active symptom monitoring
TFA: theoretical framework of acceptability
DHI: Digital health intervention
DTA: Digital Therapeutic Alliance
