# Supplementary material for: Acceptability and experience of a smartphone symptom monitoring app for people with psychosis in China (YouXin): a qualitative study
Source: BMC Psychiatry. 2024 Apr 9;24:268. doi: 10.1186/s12888-024-05687-2 (PMC11003104; doi:10.1186/s12888-024-05687-2)
Supplement: Supplementary file 1 — Supplementary Material 1: Supplementary Figure 1. Screenshots of the app prototype, Supplementary Table 2. Interview Topic guide [file 12888_2024_5687_MOESM1_ESM.docx]

**Supplementary Figure 1.** Screenshots of the app prototype: (a) the login page, (b) user centre, (c) assessment module, (e) active symptom monitoring results graph, (f) passive monitoring results graph

**Supplementary Table 1.** Demographic and clinical information for each participant

| **ID** | **Age** | **Gender** | **Ethnicity** | **Education level** | **Employment status** | **Diagnosis** | **Medication(s) used** |
| --- | --- | --- | --- | --- | --- | --- | --- |
| 001 | 34 | Male | Han | College | Working full-time | Brief psychotic disorder | Antipsychotics, anti-anxiety medications |
| 002 | 35 | Male | Han | College | Currently unemployed | Brief psychotic disorder | Antipsychotics, anti-anxiety medications |
| 003 | 59 | Male | Han | University | Working full-time | Schizophrenia | Antipsychotics, anti-anxiety medications |
| 004 | 21 | Male | Han | University | Currently unemployed | Schizophrenia | Antipsychotics, antidepressants |
| 005 | 18 | Female | Han | High school | Student | Clinical high risk | Antipsychotics, antidepressants |
| 009 | 49 | Female | Han | High school | Currently unemployed | Schizophrenia | Antipsychotics |
| 014 | 22 | Male | Han | College | Working full-time | Schizophrenia | Antipsychotics |
| 018 | 16 | Female | Han | High school | Student | Schizophrenia | Antipsychotics |
| 019 | 43 | Male | Han | College | Working full-time | Schizophrenia | Antipsychotics |
| 021 | 24 | Male | Mongolian | University | Currently unemployed | Schizophrenia | Antipsychotics |

**Supplementary Table 2.** Interview Topic guide

| **Topic** | **Questions** |
| --- | --- |
| **Introduction to using the smartphone app (YouXin):** | Please could you describe what it was like using the YouXin app?  ***Prompt:*** *Were there any things about the app that you found particularly helpful?*  ***Prompt:*** *Were there any things about the app that you didn’t find so helpful?*  How did you feel about the app when you first saw it (i.e. at the beginning of the project)?  ***Prompt:*** *When you first saw the app was there anything you particularly liked?*  ***Prompt:*** *When you first saw the app was there anything you particularly didn’t like or felt unsure about?*  Have your feelings about the YouXin app changed since your first saw the app?  ***If yes:***  ***Probe:*** *What has changed?*  What have you learned from using the app?  Has using YouXin app changed your daily life or behaviour in any way?  ***If yes:***  ***Probe:*** *what has changed?*  ***Probe:*** *how did using YouXin help with this change?*  ***If no:***  ***Probe:*** *was there anything relating to your daily life or behaviour that you had hoped YouXin might have been able to help with?*  ***Probe:*** *why do you think YouXin didn’t help you with this?*  Has using YouXin changed your thoughts and feelings about yourself in any way?  ***If yes:***  ***Probe:*** *what has changed?*  ***Probe:*** *how did using YouXin help with this change?*  ***If no:***  ***Probe:*** *was there anything relating to your thoughts and feelings about yourself that you had hoped YouXin might have been able to help with?*  ***Probe:*** *why do you think YouXin didn’t help you with this?*  Has using YouXin changed your views or understanding about psychosis in any way?  ***If yes:***  ***Probe:*** *what has changed?*  ***Probe:*** *how did using YouXin help with this change?*  ***If no:***  ***Probe:*** *was there anything relating to* your *views or understanding about that you had hoped YouXin might have been able to help with?*  ***Probe:*** *why do you think YouXin didn’t help you with this?*  Do you think YouXin has helped you?  ***If yes:*** *How?*  When we first gave you YouXin, we told you that the app is designed to help people track their experiences and symptoms over time. Do you think the app helped you with that?  ***If yes or no:***  ***Probe:*** *why do you think this?*  ***Probe:*** *how does/does it not meet this purpose?*  ***Probe:*** *what could we have included to help us meet our intended purpose? What suggestions do you have to make it better?*  Do you think this is a good way to manage symptoms for people with psychosis?  ***If yes or no:***  ***Probe:*** *why do you think this?*  Has using the app made any changes to the way you managed your mental health?  ***If yes:***  ***Probe:*** *please could you tell me more about the changes you have made and why?*  Do you think you are aware of your mood and symptoms more now than before using the phone?  ***If yes:***  ***Probe:*** *is this a good or a bad thing? Explore…*  ***Probe:*** *could you tell me more about how you are more aware than before?*  How did you feel when it came to the end of the 4-weeks using the app?  ***Prompt:*** *did you miss YouXin when you no longer had access to it?*  ***If yes:*** *in what way did you miss it?*  ***If no:*** *is there any reason why you didn’t miss having access to the app?*  Would you want to use the app for longer than 4 weeks?  ***If yes:*** *why?*  ***If no:*** *why not?*  ***Probe:*** *What is the maximum length of time you think you would want to use it for?* |
| **Completing the questions** | You were given YouXin to use over a 4-week period. Did you make any changes to how you responded to the app during the time period?  ***Probe:*** *could you tell me more about any changes in responding that you made?*  Do you feel you had enough information to help you to use the app?  ***If yes:***  ***Probe:*** *what kind of information have you valued the most?*  ***Probe:*** *what kind of information have thought was least helpful?*  ***If no:***  ***Probe:*** *what kind of information would you have found helpful and why?*  Do you find the training session at the beginning of the project helpful?  ***If yes:***  ***Probe:*** *how did it helpful?*  ***If no:***  ***Probe:*** *what could we have done to make it more useful?*  Which questions were most relevant?  ***If mention of specific questions:***  ***Probe:*** why was this aspect of the app relevant?  Was there anything missing in the app that you think we should have included?  ***Elaborate…***  Were any questions more difficult than others?  ***If yes:***  ***Probe:*** *could you tell me which ones/what made them difficult?*  ***If no:***  ***Probe:*** *were any questions easier to answer than others?*  Are there any questions you did not want to answer?  ***If yes:***  ***Probe:*** *can you give any reasons why?*  What did you think about the number of questions asked and how often they came?  ***Probe:*** *did your views on the number and how often change over time?*  What, if anything, could we have done to make it easier for you to answer the questions?  How could we make each question as meaningful for you as possible?  ***Probe:*** *did you find any questions particularly meaningful/not meaningful?*  Were the graphs/feedback helpful?  ***Probe:*** *how/why? How could we present the information in the graphs/feedback to make them more user friendly?* |
| **Passive sensing** | Did you use the passive monitoring section?  ***If yes:*** *how do you feel about being passively recorded?*  ***Probe:*** *Did you feel comfortable being passively monitored?*  ***If no:*** *why not? Was there any particular reason why you didn’t use it?* |
| **Fitting in with everyday life:** | How well did using the phone/app fit into your everyday life?  ***Probe:*** *has it changed anything that you usually do?*  Were there any times when the beeps interrupted what you were doing?  ***If yes:***  ***Probe:*** *what was it that you were doing?*  Have you shown it to anyone else or discussed it with anyone else? What were their views?  ***Prompt:*** *did you show or discuss YouXin with your psychiatrist?*  ***If yes:*** *was this helpful at all? What was their response like? Did this impact or change the care that you received, if yes, in what way?*  ***If no:*** *was there any particular reason why you didn’t discuss YouXin with your psychiatrist?*  ***Prompt:*** *did you show or discuss YouXin with any family members or friends?*  ***If yes:*** *was this helpful at all? What was their response like? Did this impact or change the care that you received, if yes, in what way?*  ***If no:*** *was there any particular reason why you didn’t discuss YouXin with family members or friends?*  If you were prompted to complete the questions whilst with other people, did you tell them about it?  ***If no:***  ***Probe:*** *what did you say instead?*  Did you feel comfortable using the app whilst there were other people around you?  ***If no:***  ***Probe:*** *Did this impact your willing to use the app?*  Did you feel that using the app ever felt part of your normal routine?  ***Probe:*** *could you tell me more about why it did/didn’t?*  How long do you think you could or would use the app for? Do you think 4 weeks is too long/not long enough?  ***Probe:*** *what would be an ideal time for you?* |
| **What could be improved:** | How could we improve the YouXin app?  ***Probe:*** *can you think of anything in terms of the way the app worked or the content of the app?*  Did you feel that the app was safe? Did you have any privacy concerns while using the app?  ***If yes:***  ***Probe:*** *what were you concerned about?*  ***Probe:*** *why did this concern you?*  Did you have any difficulties using the phone or the app at the beginning of the study?  ***Probe****: this could be a technical problem or something to do with the content?* |
| **Implementation questions:** | How can you see an app like YouXin used in the hospital?  ***Probe:*** *What would get in the way of being able to use an app like this?*  ***Probe:*** *What would help?* |
| **Any other information about YouXin not already covered:** | We’ve discussed the app in quite a lot of detail now, before we talk about your experiences of taking part in this project more generally, was there anything else you’d like to tell me specifically about your experiences of using the app? |
| **Benefits and problems:** | Now just thinking about the project more generally…  Were there any benefits to taking part in the project?  ***Probe:*** *could you tell me why they have been a benefit?*  Were there any negative consequences for taking part in the study?  ***Probe:*** *could you tell me why that has been a difficulty?*  Is there anything you would change about the process  ***If yes***  ***Probe:*** *could you tell me more about that?*  ***Probe:*** *why would you change this?* |
